# Technological Properties of Bifidobacterial Strains Shared by Mother and Child

**DOI:** 10.1155/2019/9814623

**Published:** 2019-01-17

**Authors:** Angela Peirotén, Pilar Gaya, Juan Luis Arqués, Margarita Medina, Eva Rodríguez

**Affiliations:** Departamento de Tecnología de Alimentos, INIA, Ctra. de La Coruña Km 7, 28040 Madrid, Spain

## Abstract

Technological processes in the dairy industry and the further passage through the gastrointestinal tract could impair viability and functionality of probiotic bifidobacteria. In the present work, the growth in milk of nine bifidobacterial strains shared by mother and child, their survival to freeze-drying and cold storage, and their behavior in a model cheese were investigated. All the strains exhibited high stability to the technological conditions studied when compared with two commercial strains.* Bifidobacterium breve* INIA P734 and* Bifidobacterium bifidum* INIA P671 as adjunct cultures maintained high stability during manufacture and ripening of cheese. Both strains showed, at the end of ripening period, resistance to simulated gastrointestinal conditions. Moreover, their presence did not affect negatively the quality of cheese.* B. breve* INIA P734 and* B. bifidum* INIA P671 could be considered as potential candidates for their use in cheese as adjunct cultures.

## 1. Introduction

Bifidobacteria shared by mother and infant constitute an interesting source of potential probiotic strains [1]. Among functional foods, dairy products are considered optimum vehicles for probiotics. Probiotic strains must firstly meet various technological requirements as maintenance of viability during food processing and storage, feasibility of application in products, and resistance to the physicochemical processing of foods [2]. Proteolytic activity on milk caseins, compatibility with starter cultures, tolerance to low pH of fermented foods, packaging to maintain anaerobiosis for bifidobacteria, or cold stress should also be considered. Moreover, final probiotic dairy products must have good sensory properties [3], while retaining their functionality [4, 5].

Bifidobacteria are anaerobe bacteria of intestinal origin that usually grow poorly in milk. Their viability in fermented dairy foods is a challenge to dairy processors due to the low oxidation reduction potential required for their growth, as well as their sensitivity to low pH [6]. Probiotic bifidobacterial strains should be viable at high numbers in the product at the time of consumption and minimum levels of 10^6^ cfu/g have been recommended to compensate their possible reduction after the passage through the gut [7], with dossing from 10^7^ to 10^12^ cfu [8].

Viability and functionality of a potential probiotic strain throughout the food manufacturing processes and gastrointestinal stress barriers must be monitored to guarantee that its health-promoting properties are maintained [9]. In the dairy industry, only a few strains belonging mainly to* Bifidobacterium animalis*, such as* B. animalis* BB12, are used as adjunct cultures. Some bifidobacteria have been successfully included in cheeses, since cheese pH and fat and their buffering effect may favor the protection of this microorganism over the self-life [10]. Furthermore, the cheese matrix may protect probiotic bacteria against low pH and bile salts when going through the gastrointestinal tract after consumption [11, 12]. Thus, many studies have been conducted with commercial strains in different cheese varieties [13–17].

In the present work, technological properties of nine mother-infant shared* Bifidobacterium *spp. strains [18] were investigated. Model cheeses with two selected bifidobacterial strains as adjunct cultures were elaborated to evaluate their impact in cheese quality and their survival at the end of the ripening period to a digestive* in vitro* assay.

## 2. Materials and Methods

### 2.1. Bacterial Strains and Culture Conditions

Nine bifidobacterial strains previously settled to be shared by mother-infant pairs [18] were selected. The commercial probiotic strains* B. animalis* BB12 (Chr. Hansen A/S, Hørsholm, Denmark) and* Bifidobacterium longum *BB536 (isolated from a Morinaga product) were used for comparative purposes. Strains were routinely cultured in RCM broth (Becton, Dickinson and Company, Franklin Lakes, NJ), incubated at 37°C for 48 h in anaerobic atmosphere (AnaeroGen, Oxoid, Basingstoke, UK), and maintained at -80°C in RCM with 15% (v/v) glycerol. Isolates were subcultured twice on RCM agar before their use in subsequent experiments.

### 2.2. Technological Properties of Shared Bifidobacterial Strains

Survival as frozen cultures was measured after 21 days of storage at -80°C. The strains were grown in RCM for 48 h at 37° in anaerobic conditions and glycerol was added as a cryopreservant to a final concentration of 5% (w/v). Viable cell population was determined by plate counting on RCM agar before and after the storage at -80°C.

In order to test their viability as lyophilized cultures, cells were in the first place resuspended in skimmed milk (10%) (Central Lechera Asturiana, Siero, Spain) as protective medium and frozen at -80°C for 24 h. Subsequently, cultures were lyophilized in a Cryodos model lyophilizer (Telstar S.A., Terrasa, Spain) operating at 1 Pa pressure and -45°C for 24 h. Lyophilized cultures were stored at 5°C for 21 days. Freeze-dried cells were reconstituted using peptone water and viability was determined by plate counting on RCM agar.

The ability to grow in milk was tested in reconstituted skimmed milk (10% w/v) inoculated with bifidobacterial cultures and incubated in anaerobic conditions at 37°C for 24 h. Counts were assessed by plate counting on RCM agar at 0 and 24 h.

Viability in milk under refrigerated storage was tested in reconstituted skimmed milk inoculated with bifidobacterial cultures and stored at 5°C. Viable cell population was determined at 0, 14, and 28 days by plate counting on RCM agar.

### 2.3. Behavior of Bifidobacteria in Model Cheeses

#### 2.3.1. Cheese Manufacture

Semihard model cheeses inoculated with* B. breve* INIA P734 and* B. bifidum* INIA P671 were manufactured from pasteurized cow's milk in duplicate experiments. Three vats of 2 L of milk each were processed each day: vat 1 (control) without bifidobacterial strains, vat 2 with* B. breve* INIA P734, and vat 3 with* B. bifidum* INIA P671. Bifidobacteria were resuspended in pasteurized cow's milk (approximately 7-8 log cfu/ml milk) and added to the correspondent vat at 1%, after the addition of the commercial starter MA 016. Cheeses were made according to Gómez-Torres et al. [19]. One cheese, of approximately 200 g in weight, was obtained from each vat. Cheeses were pressed overnight at 20°C, vacuum-packaged in Cryovac plastic bags, and ripened at 12°C for 28 d.

#### 2.3.2. Microbial Determinations

Microbiological determinations were carried out at days 1, 7, 15, and 28. Cheese samples (5 g) were homogenized with 45 ml of a sterile 2% (w/v) sodium citrate solution at 45°C and decimal dilutions were prepared in a sterile 0.1% (w/v) peptone solution. Bifidobacterial counts were determined on duplicate plates of RCM agar supplemented with 50 mg/l mupirocin (Oxoid, Basingstoke, UK) (RCA-MUP) and incubated at 37°C for 48 h under anaerobic conditions. Lactococci counts from the commercial starter were determined in milk and cheese samples on M17 agar (Biolife) supplemented with glucose at 0.5 g/ml (GM17) and incubated at 40°C and 30°C for 24 h, and total counts on PCA agar (Biolife) for 24 h at 30°C.

#### 2.3.3. Chemical Determinations

Cheese pH was measured in duplicate by means of a Crison pH meter (model GPL 22, Crison Instruments, Barcelona, Spain) using a Crison penetration electrode (model 52-3.2).

Cheeses overall proteolysis was determined on duplicate samples by the* o*-phthaldialdehyde (OPA) test as described by Church et al. [20].

Sugars and organic acids were extracted from duplicate samples of cheese and determined by high-performance liquid chromatography (HPLC) [21]. Extracts (50 *μ*l) were injected in duplicate and eluted with 3 mM sulfuric acid at 65°C and a flow rate of 0.7 ml/min on a 300 x 7.8 mm Aminex HPX-87H ion exchange column protected by a cation H+ Micro-Guard cartridge (Bio-Rad Laboratories, Richmond, CA, USA) in a Beckman System Gold-Liquid Chromatograph (Beckman Instruments S.A., Madrid, Spain), equipped with two detectors connected in series, a diode array detector with a detection wavelength of 210 nm for citric, pyruvic, lactic, acetic, propionic, and butyric acids and at 280 nm for orotic and uric acids, and a differential refractometer detector module (Knauer, Berlin, Germany) for sugars (lactose, glucose, and galactose). Organic acids and sugars were quantified by the external standard method. The results were expressed as micrograms per gram of cheese.

Volatile compounds in 28 d cheeses were extracted by automated solid-phase microextraction (SPME) and analyzed by gas chromatography-mass spectrometry (GC-MS) (HP 6890-MSD HP 5973, Agilent Technologies, Santa Clara, CA). Duplicate 10 g cheese samples were homogenized in an analytical grinder (IKA, Labortechnik, Staufen, Germany), with 20 g of anhydrous Na_2_SO_4_ and 50 *μ*l of an aqueous solution of 1 mg/ml cyclohexanone (Merck KGaA, Darmstadt, Germany) as internal standard (IS). Two grams of this mixture was weighed in a 20 ml headspace glass vial sealed with a polytetrafluorethylene (PTFE) faced silicone septum (Agilent Technologies). The equilibration (37°C/20 min), extraction (37°C/30 min), and injection and desorption (260°C/10 min in splitless mode) phases were carried out using a CTC CombiPAL autosampler (Agilent Technologies). The volatile compounds were extracted using a 2 cm x 50/30 *μ*m StableFlex Divinylbenzene/Carboxen/Polydimethylsiloxane (DVB/CAR/PDMS) coated fiber (Supelco, Bellefonte, PA). Chromatographic separation was carried out in a Zebron 100% polyethylene glycol capillary column (60 m long; 0.25 mm internal diameter; 0.50 *μ*m film thickness; ZB-WAXplus, Phenomenex, Torrance, CA). Initial helium flow was 1.4 ml/min kept for 1 min, 1 ml/min helium flow, with the following temperature program: 7 min at 40°C, first ramp 2°C/min to 90°C, second ramp at 3°C/min to 150°C, and a final ramp to 240°C at 9°C/min. Mass detection was performed in the scan mode, from 33 to 280 amu at 5.53 scan/s and ionization by EI at 70 eV. Volatile compounds were identified by comparison of spectra with the Wiley7Nist05 library (Wiley and Sons Inc., Germany) and by comparison of their retention times with authentic standards (Merck). Relative abundances of compounds were expressed as percentages of peak areas to the IS peak area. Samples were tested in duplicate.

### 2.4. Survival of Bifidobacteria Vehiculized in Cheese to Simulated Gastrointestinal Conditions

Cheese samples (5 g) from 28 d cheeses were diluted in 45 ml of acid solution (Phosphate Buffered Saline, PBS; pH 3) at 37°C and homogenized for 90 s in a stomacher. Homogenates were incubated at 37°C under anaerobic conditions for one hour. Subsequently, 1 ml was taken, added to 9 ml bile solution (0.15%, Ox-bile desiccated, Oxoid), and kept at the same conditions for 1 h. Counts were made on duplicate plates of RCA-MUP to test the survival of bifidobacterial strains. Survival of the starter culture in control cheese was examined on GM17 agar.

### 2.5. Statistical Analysis

Statistical treatment of data was performed by means of SPSS Statistics 22.0 software (IBM Corp., Armonk, NY, USA). Data were analyzed by ANOVA using a general linear model. Comparison of means was carried out by Tukey's test or Dunnett's test for a confidence interval of 99%.

## 3. Results and Discussion

### 3.1. Technological Properties of Bifidobacterial Strains

Technological properties of nine bifidobacterial strains, selected due to being shared by mother and child, were tested for their further use in dairy products as potential probiotic adjunct cultures. Two commercial bifidobacterial strains were included for comparative purposes. Good stabilities were observed among bifidobacteria studied as frozen or freeze-dried cultures (Table 1), although differences for a given strain were found for both traits (*P*<0.01).

The best stability was found in* B. animalis* BB12. The viability of the freeze-dried microorganisms depends on several factors such as strain, bacterial cell size, and efficacy of the cryoprotectant [22]. In this regard, du Toit et al. [8] described not only viability, but also different probiotic properties of the same strains as fresh, freeze-dried, fresh heat-tolerant, and freeze-dried heat-tolerant strains.

The growth of bifidobacteria in milk or dairy products is limited compared with lactic acid bacteria used in fermented dairy products [23]. Concerning the ability of the selected bifidobacterial strains to grow in milk (Table 2), seven out of the nine strains grew in milk without any added growth factor, with increases of 1-2 log cfu in four strains.

Viability of bifidobacteria in dairy products during cold storage is a main concern in developing new probiotic formulations. Refrigeration in milk resulted in variable reductions in numbers over storage (Table 2).* B. animalis* BB12 showed the best stability under refrigeration. After 14 d of storage, reductions higher than 1 log unit were observed for* B. bifidum *INIA P826,* B. longum* BB536,* B. infantis* INIA P737, and* B. breve* INIA P712. After 28 d, further diminutions were observed. In* B. breve* INIA P712 and the commercial strain* B. longum* BB536 the decrease during refrigerated storage was higher than that observed with other storage processes (Tables 1 and 2). This could be related to a higher tolerance induced by the previous freeze-drying stress on cells [24]. Viability of strains in fermented milk has been linked to acidification, oxide-reduction potential, and relative fatty acid composition, which differ among the strains and the types of milk used [25]. In the present work, cells suspensions were maintained in milk without any protective compound and under aerobic conditions. Bifidobacterial strains are strictly anaerobic, and reductions of viability during refrigeration could be mainly attributed to redox potential. Different protective strategies like the addition of L-cysteine [26], acid casein hydrolysate, whey proteins or tryptone in yogurt [27], resistant starch [28], gases [29], or encapsulation [30] have been proposed.

According to our results, the ability to grow in milk was not linked to their resistance to cold storage at 4°C. Only* B. bifidum* INIA P671 grew in milk and after 28 d of cold storage showed reductions lower than 0.3 log units. Cold storage in milk and freeze-drying may play a crucial role in strain survival during product storage. Thus, strains with technological abilities in between the two commercial strains tested were selected for further studies.

### 3.2. Behavior of B. breve INIA P734 and B. bifidum INIA P671 in Cheese Models

The most popular food delivery systems for probiotic cultures have been fermented milks. However, cheese may be more effective than yogurt-like products in delivering probiotic bacteria to the intestinal tract [23]. Considering the results obtained after freeze-drying followed by refrigeration, and their ability to grow in milk,* B. breve* INIA P734 and* B. bifidum* INIA P671 were selected as adjunct cultures for cheese making.

#### 3.2.1. Microbial Determinations

The starter culture used in the present work was not affected by the use of bifidobacteria as adjunct cultures. Counts of total viable bacteria in all cheeses remained at levels >9.5 log cfu/g during the 28 d ripening period, with no significant differences between cheeses with bifidobacteria and control cheese (*P*<0.01) (data not shown).* B. breve* INIA P734 and* B. bifidum* INIA P671 presented good stability after cheese making and through ripening (Table 3). Both strains survived cheese making and their levels increased by more than 1 log unit, probably due to cell entrapment in pressed curd. Bifidobacterial counts remained stable (*P*<0.01) during cheese ripening, exhibiting good survival comparable to that reported for* B. animalis *BB12 in cheddar cheese [13, 31].

In cheddar cheese made with buffalo milk with* Lactobacillus acidophilus* LA-5,* B. bifidum* Bb-11 and* B. longum* BB536, levels > 7 log cfu/g were reached through the 180 d of ripening [10]. On the contrary,* B. longum* BB536 counts declined from approximately 8 log cfu/g to 5 log cfu/g after a standard cheddar cheese making protocol and the same time of ripening [13]. Similar results were also recoded for two strains of* B. animalis*, Bf26 and Bf141, in low-fat cheddar [32] and* B. longum *DJO10A in cheddar cheese [31].

#### 3.2.2. Chemical Determinations

Probiotic cultures should not modify negatively the sensorial properties of the cheese to which they are added. These cultures can induce changes in the chemical composition and the texture; however, they do not necessarily have a noticeable effect on flavor [33]. In the present work, cheese pH values (data not shown) showed minor differences (*P*<0.01) between cheeses that were unrelated to the addition of bifidobacteria. Consequently, the starter culture was not affected by the addition of the bifidobacterial strains in terms of milk fermentation.

Citric, pyruvic, lactic, and acetic acids were detected in all the cheeses throughout ripening (Table 4). Citric content increased in cheeses with* B. bifidum* INIA P671 compared to control cheese (*P*<0.01) but not in cheese with* B. breve* INIA P734, which exhibited a significant increase of acetic acid over ripening higher than in control cheese. Lactic acid content was lower in both cheeses than in control. Sugars (lactose, glucose, and galactose) were not detected.

Overall proteolysis (OPA test) in 28 d cheeses was not affected by the addition of bifidobacterial adjuncts. Values for absorbance at 340 nm were 0.47 ± 0.02 in control cheese, 0.57 ± 0.08 in cheese with INIA P734, and 0.55 ± 0.03 in cheese with INIA P671, without significant (*P*<0.01) differences among them.

In the volatile fraction, 29 compounds (3 carboxylic acids, 2 esters, 5 ketones, 10 terpenes, 2 hydrocarbons, 2 benzene compounds, and 5 miscellaneous compounds) were identified. Thirteen compounds showed significant (*P*<0.01) differences in cheeses made with bifidobacteria with respect to control cheese (Table 5) at the end of the ripening period. Carbon disulfide, acetic and butanoic acids, their ethyl-esters, diacetyl and acetoin were higher for one or both bifidobacterial cheeses with respect to control cheeses. Higher acetic acid values could be attributed to the metabolic activity of bifidobacteria through the fructose-6-phosphate shunt pathway utilizing residual lactose [34]. Acetic acid is detected at high concentrations in similar cheeses, like cheddar, and normally contributes to its flavor, although very high concentrations could produce off flavors [35].

Acetoin was higher in both cheeses with bifidobacteria. It has been reported that bifidobacteria may convert pyruvate to acetoin instead of organic acids to maintain their internal pH [36]. The increased production of acetoin, 2-butanone, and acetic acid has been correlated with a higher yield of ATP for supporting the pH homeostasis by F_1_F_0_ATPase [37, 38].

Overall, the GC-MS results indicated that the bifidobacterial strains had a considerable effect on the generation of esters and ketones throughout ripening that can contribute to cheese flavor.

### 3.3. Survival of Bifidobacteria and Starter Culture in Cheese to Simulated Gastrointestinal Conditions

Cheese can act as a buffer against the high acidic conditions of the gastrointestinal tract, favoring probiotic survival. Moreover, its high fat content may offer additional protection to probiotic bacteria during passage through the gastrointestinal tract [39, 40]. An essential step towards the selection of potential probiotic candidates is to examine their resistance under GI stress conditions [9]. Here, cheeses with bifidobacteria and control cheese were submitted at the end of the ripening to a simulated gastrointestinal passage.

In the present study, the starter culture survived in control cheeses homogenates at high levels, with counts of 9.42 ± 0.09 log cfu/g before digestion and 8.75 ± 0.15 cfu/g after digestion. Consequently, bacteria from the starter culture might arrive at high levels to the intestine where they could play a beneficial role. Simulated digestion resulted in a decrease in the viability of approximately 1.7 log units for the bifidobacterial strains, with final counts of* B. breve* INIA P734 and* B. infantis* INIA P671 of 6.55 ± 1.26 and 4.96 ± 0.16 log cfu/g log cfu/g, respectively. Tolerance of probiotic bacteria to acid and bile has been described as variable and strain dependent [41]. Moreover, a protective effect of milk or milk components against low pH has been reported in both lactic acid bacteria and bifidobacteria [42, 43]. In this aspect, the acid tolerance of probiotics seems to be better in milk and cheese than in PBS or yogurt, respectively [44, 45]. Milk fat, solid consistency, or buffering capacity of the food matrix may be involved in the protective effect observed on bifidobacterial survival during* in vitro* gastrointestinal challenge studies [16, 46]. According to our results, values around 5-6.5 log units after simulated digestion would lead to 7-8.5 log cfu for both bifidobacterial strains after the ingestion of 100 g of ripened cheese. In consequence, this study shows the suitability of semihard cheese as a vehicle for delivery of these two bifidobacterial strains.

## 4. Conclusions

Technological properties were recorded for nine bifidobacterial strains shared by mother and child. The selected strains,* B. breve *INIA P734 and* B. bifidum *INIA P671, survived cheese making and the ripening period, not affecting the cheese quality. Moreover, these bifidobacteria vehiculated in the 28 d ripened cheeses showed good resistance to the simulated gastrointestinal conditions, suggesting that they may survive through the human GI transit at acceptable levels. In conclusion,* B. breve *INIA P734 and* B. bifidum *INIA P671 were considered good candidates as adjunct cultures in semihard cheeses as a vehicle of potential probiotics.

## Figures and Tables

**Table 1:** Changes (log cfu/ml) in bifidobacterial counts after freezing or freeze-drying and subsequent storage.

Strain	-80°C (21 d)	Freeze-drying (21 d)
*B. breve *INIA P712	-0.99 ± 0.58^ab^	-1.76 ± 0.18^a^
*B. longum *subsp.* longum *INIA P678	-0.67 ± 0.17^de^	-1.10 ± 0.09^bc^
*B. breve *INIA P734	-0.95 ± 0.07^abc^	-0.10 ± 0.03^e^
*B. longum *subsp.* infantis *INIA P737	-0.63 ± 0.31^de^	-0.85 ± 0.26^cd^
*B. bifidum *INIA P671	-0.58 ± 0.05^de^	-0.95 ± 0.07^bcd^
*B. pseudocatenulatum *INIA P753	-1.12 ± 0.18^a^	-0.65 ± 0.26^d^
*B. adolescentis *INIA P784	-0.10 ± 0.05^f^	-1.20 ± 0.14^b^
*B. bifidum *INIA P826	-0.81 ± 0.10^bcd^	-0.99 ± 0.22^bc^
*B. longum *subsp.* longum *INIA P843	-0.75 ± 0.08^cde^	-0.86 ± 0.22^cd^
*B. animalis *BB12	-0.13 ± 0.06^f^	-0.16 ± 0.08^e^
*B. longum *subsp.* longum *BB536	-0.53 ± 0.09^e^	-1.65 ± 0.23^a^

Values are the mean ± SD (n=4). Means in the same column with a different superscript differ significantly (*P*<0.01).

**Table 2:** Growth in milk (log cfu/ml) and changes in bifidobacterial strains during subsequent storage at 4°C.

Strain	Growth in milk	Storage at 4°C	Storage at 4°C
	(1 d)	(14 d)	(28 d)
*B. breve *INIA P712	1.45 ± 0.20^e^	-3.60 ± 0.22^a^	-
*B. longum *subsp.* longum *INIA P678	0.85 ± 0.06^d^	-0.68 ± 0.05^d^	-1.00 ± 0.02^c^
*B. breve *INIA P734	0.42 ± 0.22^c^	-0.69 ± 0.10^d^	-1.85 ± 0.03^b^
*B. longum *subsp.* infantis *INIA P737	1.88 ± 0.15^f^	-1.58 ± 0.15^b^	-1.94 ± 0.06^b^
*B. bifidum *INIA P671	1.29 ± 0.10^e^	-0.21 ± 0.15^e^	-0.28 ± 0.25^de^
*B. pseudocatenulatum *INIA P753	-0.38 ± 0.23^b^	-0.09 ± 0.12^e^	-0.34 ± 0.16^d^
*B. adolescentis *INIA P784	-1.44 ± 0.17^a^	-0.59 ± 0.12^d^	-0.41 ± 0.19^d^
*B. bifidum *INIA P826	1.54 ± 0.55^e^	-1.04 ± 0.37^c^	-1.19 ± 0.72^c^
*B. longum *subsp.* longum *INIA P843	0.53 ± 0.15^c^	-0.85 ± 0.10^cd^	-1.62 ± 0.11^b^
*B. animalis *BB12	0.44 ± 0.04^c^	-0.13 ± 0.10^e^	-0.05 ± 0.11^e^
*B. longum *subsp.* longum *BB536	0.91 ± 0.16^d^	-1.17 ± 0.07^c^	-2.39 ± 0.07^a^

Values are the mean ± SD (n=4). Means in the same column with a different superscript differ significantly (*P*<0.01).

**Table 3:** Bifidobacterial counts (log cfu/g) in cheeses made with *B. breve* INIA P734 or *B. bifidum* INIA P671 as adjunct culture.

Strain	1 d	7 d	15 d	28 d
Control	ND	ND	ND	ND
*B. breve* INIA P734	8.44 ± 0.32^a^	8.55 ± 0.15^a^	8.50 ± 0.29^a^	8.27 ± 0.39^a^
*B. bifidum* INIA P671	7.10 ± 0.11^a^	7.13 ± 0.19^a^	6.94 ± 0.27^a^	6.61 ± 0.34^a^

Values are the mean ± SD (n=4). Means in the same row with the same superscript do not differ significantly (*P*<0.01).

**Table 4:** Organic acids in cheeses with bifidobacteria and control cheese.

Organic acid	Ripening (days)	Control cheese	*B. breve* INIA P734	*B. bifidum* INIA P671
Acetic acid	1	944.81 ± 16.03	1049.32 ± 136.62^*∗*^	879.06 ± 26.00^*∗*^
	15	1040.58 ± 25.59	1150.45 ± 162.72^*∗*^	968.51 ± 36.69^*∗*^
	30	1109.49 ± 52.71	1232.27 ± 108.90^*∗*^	1079.71 ± 77.67
Citric acid	1	1539.38 ± 75.12	1428.70 ± 107.12^*∗*^	1638.72 ± 11.55^*∗*^
	15	1712.67 ± 4.87	1577.84 ± 43.60^*∗*^	2145.82 ± 117.08^*∗*^
	30	1774.38 ± 32.94	1719.40 ± 111.78^*∗*^	2465.29 ± 23.82^*∗*^
Lactic acid	1	19650.38 ± 494.24	15965.66 ± 2051.09^*∗*^	16145.76 ± 602.30^*∗*^
	15	19718.31 ± 1492.70	15841.27 ± 1410.43^*∗*^	16916.74 ± 431.25^*∗*^
	30	18869.98 ± 1499.33	16084.38 ± 2180.85^*∗*^	16963.25 ± 1033.72^*∗*^
Pyruvic acid	1	111.26 ± 15.95	132.94 ± 11.13^*∗*^	151.05 ± 7.35^*∗*^
	15	112.36 ± 7.19	116.10 ± 3.64^*∗*^	154.28 ± 14.54^*∗*^
	30	124.02 ± 3.56	123.65 ± 21.41	170.84 ± 18.62^*∗*^

Values are presented as the mean ± SD (n=4) and expressed as micrograms of acid per gram of cheese. Means with ∗ in the same row differ significantly (*P*<0.01, Dunnett's test) from the control cheese.

**Table 5:** Volatile compounds (with significant differences) in model cheeses made with *B. breve* INIA P734 and *B. bifidum* INIA P671 as adjunct cultures.

	QI	Control	*B. breve* INIA P734	*B. bifidum *INIA P671
**Miscellaneous**				
Acetaldehyde	44	1.75 ± 0.04	1.41 ± 0.33^*∗*^	1.05 ± 0.37^*∗*^
Carbon disulfide	TIC	22.60 ± 10.39	51.41 ± 3.51^*∗*^	38.68 ± 11.88^*∗*^
Dimethyl sulfide	TIC	7.05 ± 2.78	4.35 ± 0.85^*∗*^	5.27 ± 1.69
Ethene, trichloro-	130, 95, 60	ND	0.99 ± 0.25^*∗*^	ND
**Carboxylic acids**				
Acetic acid	43, 45, 60	75.63 ± 7.55	235.32 ± 153.49^*∗*^	89.79 ± 39.04
Butanoic acid	TIC	62.93 ± 4.35	131.73 ± 56.99^*∗*^	70.73 ± 13.61
**Esters**				
Ethyl acetate	TIC	3.50 ± 0.45	10.22 ± 7.50^*∗*^	5.41 ± 0.99^*∗*^
Ethyl butanoate	71, 88, 60, 101	2.28 ± 0.58	2.42 ± 0.21	2.70 ± 0.36^*∗*^
**Ketones**				
2-Propanone (acetone)	TIC	42.59 ± 10.55	30.38 ± 4.09^*∗*^	31.64 ± 8.95^*∗*^
2-Butanone	TIC	24.43 ± 2.90	19.63 ± 0.99^*∗*^	21.03 ± 2.85^*∗*^
2,3-Butanedione (diacetyl)	43, 86	15.81 ± 4.09	29.88 ± 8.73^*∗*^	30.82 ± 0.82^*∗*^
3-Hydroxy-2-butanone (acetoin)	43, 45, 88	99.75 ± 61.24	244.38 ± 121.52^*∗*^	209.68 ± 22.27^*∗*^
**Terpenes**				
Camphane	95, 81, 123	1.23 ± 0.05	1.06 ± 0.14^*∗*^	1.01 ± 0.13^*∗*^

Volatile compounds levels are expressed as relative abundances to the internal standard cyclohexanone calculated from (peak area/IS peak area) × IS concentration. (IS= cyclohexanone; IS concentration=500 *μ*g per mg of cheese). TIC, total ion count; ND, not detected. Means with *∗* in the same row differ significantly from control cheese according to Dunnett's test (*P* < 0.01).

## Data Availability

The data used to support the findings of this study are available from the corresponding author upon request.
